# Quantitative genetic analyses of traits related to calcium and phosphorus metabolism in two different laying hen strains

**DOI:** 10.1186/s12711-026-01066-5

**Published:** 2026-07-21

**Authors:** Valentin P. Haas, Markus Schmid, Vera Sommerfeld, Amélia Camarinha-Silva, Martina Feger, Michael Föller, Korinna Huber, Michael Oster, Siriluck Ponsuksili, Jana Seifert, Klaus Wimmers, Jörn Bennewitz, Markus Rodehutscord

**Affiliations:** 1 Institute of Animal Science, University of Hohenheim, Stuttgart, 70599 Germany; 2 HoLMiR - Hohenheim Center for Livestock Microbiome Research, University of Hohenheim, Stuttgart, 70599 Germany; 3 Department of Physiology, University of Hohenheim, Stuttgart, 70599 Germany; 4 Research Institute for Farm Animal Biology (FBN), Dummerstorf, 18196 Germany; 5 Faculty for Agriculture, Civil and Environmental Engineering, University of Rostock, Rostock, 18059 Germany

**Keywords:** Mineral bioavailability, Feed efficiency, *Myo*-inositol, Phytate degradation, Heritabilities, Phosphorus utilization

## Abstract

**Background:**

Phosphorus (P) and calcium (Ca) are essential minerals for laying hens. Phosphorus in plant feeds is mainly stored as phytate and needs to be released by the enzyme phytase. Due to the high requirement of Ca, laying hens exhibit limited endogenous phytate degradation and thus plant-P is available to a limited extent. Mineral P supplemented to laying hen feed reduces phytate degradation further and decreases *myo*-inositol release in the intestinal tract, which is known to have many functions in poultry metabolism. The focus of this study was the investigation of P and Ca metabolism in the peak period of egg production in commercial hybrid laying hens from Lohmann Selected Leghorn (LSL; *n* = 200) and Lohmann Brown (LB; *n* = 200) strains at the phenotypic and quantitative genetic level using data referring to blood plasma, ileal digesta, excreta, and eggs.

**Results:**

Population genetic analyses revealed larger genetic diversity in LB than LSL and substantial differentiation between the strains. The majority of Ca and P metabolism traits differed significantly in trait mean or variance between the two strains. The LB strain showed more trait variation at the phenotypic and quantitative genetic levels. Moderate to high and significant heritabilities were estimated for *myo*-inositol in the plasma ($\:h^2$ = 0.43 for LSL and $\:h^2$ = 0.36 for LB), ileum digesta ($\:h^2$ = 0.60 for LB; not estimable for LSL) and egg ($\:h^2$ = 0.69 for LSL and $\:h^2$ = 0.55 for LB), and for the Ca concentration in the plasma ($\:h^2$ = 0.27 for LB; not estimable for LSL). Noticeably significant phenotypic correlations between the traits of P and Ca metabolism measured in excreta, plasma, and ileal digesta were present in both strains.

**Conclusion:**

The study provided a comprehensive insight into P and Ca metabolism under standardized conditions in the two commercial laying hen strains LSL and LB during egg laying. Differences between the strains were present at the phenotypic and quantitative genetic level. Thereby, the hens’ genetics appeared to be a relevant driver of P and Ca metabolism, with LB showing more variability. The study confirmed population genetic differences between the strains. Despite the detected strain differences, significant correlations among the traits of P and Ca metabolism indicate that the general relationships between traits are comparable in both strains.

**Supplementary Information:**

The online version contains supplementary material available at 10.1186/s12711-026-01066-5.

## Background

Besides calcium (Ca), phosphorus (P) is an essential mineral for laying hens. In typical layer diets, Ca is supplied mainly from mineral sources (e.g., limestone), whereas most P inherent to plant ingredients is bound as phytate, the salt of *myo*-inositol 1,2,3,4,5,6-hexakis phosphate (InsP_6_), and therefore requires phytate-degrading enzymes (phytase and related phosphatases) for bioavailability [1]. Poultry in general, and laying hens in particular, have limited endogenous phytase activity in the digestive tract due to their high Ca requirement [1–3]. Calcium is known to decrease phytate degradation either by directly inhibiting phosphatases or by formation of less soluble Ca-phytate chelates [4]. Thus, plant-P is not available to a large extent. Therefore, conventional poultry diets are widely supplemented with exogenous microbial phytase or mineral P sources, or both. Supplementation with mineral P leads to even more reduced phytate degradation [5] and subsequent increased P excretion, potentially with an adverse environmental impact [6]. Besides phytate degradation, dietary P supplementation can decrease *myo*-inositol (MI) release in the intestinal tract, the final product of intestinal phytate cleavage. *Myo*-inositol is known for its many functions in animal metabolism [7]. In poultry, dietary MI affects several physiological systems, for example glucose and insulin metabolism, bone mineralization, growth performance, and neural and neuroendocrine functions [8]. Differences were found between brown and white laying hen strains regarding MI concentrations in the small intestine, egg yolk, and blood plasma, suggesting distinctive metabolisms [3, 9]. Endogenous phosphatase activity varies among individuals, and so does the ability to degrade phytate and to utilize the released P. This is in line with great interindividual variations for various phenotypes related to P utilization in laying hens [10–12], the moderate heritability found for P utilization in Japanese quail [13], and phytate P bioavailability in broilers and layers [14, 15]. In addition, Vollmar et al. [16] found quantitative trait loci (QTL) regions for P utilization and strongly correlated efficiency traits in Japanese quail. Despite comparable performance levels, there are differences between commercial laying hen strains, such as Lohmann Selected Leghorn (LSL) and Lohmann Brown (LB), at the phenotypic level during maturation [17] and metabolism during the laying period [18], at the genomic level [19, 20], as well as other levels of the omics cascade [21, 22]. Some results of comparative studies point to differences in Ca and P metabolism between these two commercial layer strains (e.g., [18, 23]). A more detailed view of the traits of this complex has previously demonstrated that LB hens differ from LSL hens with regard to P and Ca metabolism-related traits like transporter expression [11] and blood hormones [23, 24], MI concentrations in blood and eggs [3], and kidney MI oxygenase (MIOX), a key enzyme in MI catabolism [25].

The present study investigated complex traits of P and Ca metabolism during the peak period of egg production in commercial hybrid laying hens from LSL and LB at the phenotypic and quantitative genetic level using samples of blood plasma, ileal digesta, excreta, and eggs, providing a novel, comprehensive insight into P and Ca metabolism. In order to avoid potential genetic effects on mineral metabolism and excretion being confounded by surplus P intake, the experimental design aimed at a low P supply for the hens. The traits of P and Ca metabolism were analyzed along with feed intake and laying performance of the hens to comprehensively place these traits in context.

## Materials and methods

This study was part of the interdisciplinary Research Unit P-Fowl: Inositol phosphates and MI in the domestic fowl: Exploring the interface of genetics, physiology, microbiome, and nutrition (https://p-fowl.uni-hohenheim.de). The animal trial was conducted at the Agricultural Experiment Station of the University of Hohenheim, Germany, following approval by the Regierungspräsidium Tübingen (regional administrative authority in the state of Baden-Württemberg), Germany (Project no. HOH67-21TE), in accordance with the German Animal Welfare Legislation.

### Experimental population and housing

The experimental population consisted of 200 LB and 200 LSL commercial hybrid hens that descended from 10 unrelated roosters per strain, representing two distinct genetic backgrounds. The hatchlings were obtained from the breeding company Lohmann Breeders GmbH, Cuxhaven, Germany. They were kept at the Research Institute for Farm Animal Biology, Dummerstorf, Germany, where they were raised in floor pens on deep litter bedding according to the standardized routine procedure of the station, including a complete vaccination program. At 7 weeks of age, hens were transferred to the Agricultural Experiment Station of the University of Hohenheim (Unterer Lindenhof, Eningen, Germany) and further raised according to a standard protocol. After jointly rearing all animals, the experiment was conducted cohort-wise in four consecutive experimental periods, when the animals were 27, 31, 35, and 39 weeks old, respectively, due to limitations in experimental capacity. Within each cohort, offsprings from all sires per strain were evenly distributed and randomly assigned to metabolic units. The same diet, based on corn and soybean meal without exogenous phytases and without mineral P supply, was fed throughout the entire experiment to ensure standardized feeding across cohorts. For each experimental period, a cohort of five offspring from each rooster was selected per strain and kept in individual metabolic units (single unit, 1 m × 1 m) for three weeks. Thereby, hens were maintained under a 16-hour light and 8-hour dark cycle, with barn temperatures set between 18 and 22 °C. Each individual metabolic unit was equipped with a wooden perch, a nest, a feeding trough, water cups, and a wire mesh floor. For total excreta collection, stainless-steel trays were placed underneath the units.

### Experimental diet

The experimental diet was based on corn and soybean meal to minimize plant intrinsic phytase activity (Additional file 1: Table S1). The diet was calculated to contain adequate levels of all nutrients according to the recommendations of the Society of Nutrition Physiology (Gesellschaft für Ernährungsphysiologie, GfE) [26], except for P. It contained TiO_2_ as an indigestible marker and was provided in mash form. Exogenous phytases and mineral P were not added. Diet components were mixed in the certified feed mill of the Agricultural Experiment Station of the University of Hohenheim. Feed and water were provided for *ad libitum* consumption. The calculated nutrient concentrations were confirmed by analyses overall (Additional file 1: Table S2).

### Experimental sampling procedures, phenotyping, and genotyping

**Fig. 1:**
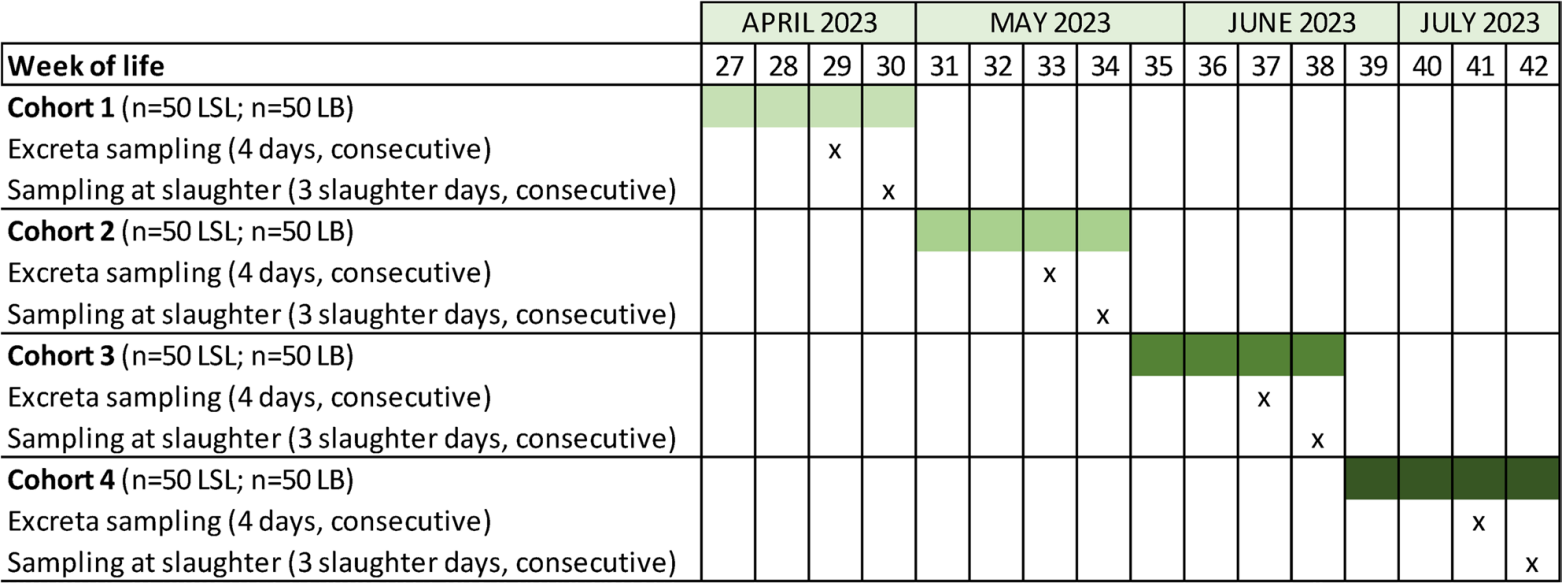
Sampling timeline to investigate Ca and P metabolism traits in Lohmann Selected Leghorn (LSL) and Lohmann Brown (LB) laying hens

The timeline of the sampling procedures is given in Fig. 1. All procedures were standardized across the four experimental periods. Each experimental period was characterized by a habituation phase of two weeks, subsequent excreta sampling for four consecutive days (days 15–18 of the period), and three consecutive slaughter days in week 4 (days 22, 23, and 24 of the period). Accordingly, depending on the cohort to which they belonged, the animals were 29, 33, 37, and 41 weeks old at excreta sampling and 30, 34, 38, and 42 weeks old at slaughter. Since the offsprings of all sires per strain were randomly represented in the metabolic units within each cohort, the individual slaughter days included animals from each sire. Animals in the metabolic units were inspected at least twice a day to monitor clinical signs and ensure their health and welfare. The eggs of all hens were collected and weighed over the three weeks of habituation and excreta sampling. The hens were weighed at the beginning of the sampling period, during excreta sampling, and on the day of slaughtering. Feed was weighed at the beginning and end of each sampling period. Total excreta were collected from the trays at 24-h intervals for four consecutive days in the morning hours. Feathers, skin scales, and spilled feed were carefully removed from the trays before every collection, and the excreta were immediately frozen at -20 °C. The leftovers from the trays and troughs were weighed, and feed intake was adjusted accordingly. Before slaughtering, the hens were deprived of feed for 1 h, followed by 1 h of *ad libitum* access to feed to standardize gut fill. The hens were individually stunned with a gas mixture of 35% CO_2_, 35% N_2_, and 30% O_2_ and killed by bleeding. Trunk blood was collected in tubes containing lithium heparin for P and Ca analysis. Blood samples were centrifuged for 10 min at 2,500 × *g* to obtain plasma. Plasma was stored at -80 °C until analysis. Digesta from the terminal part of the ileum, defined as the posterior two-thirds of the section between Meckel´s diverticulum and 2 cm prior to the ileo-ceco-colonic junction, were collected by gently squeezing. Digesta samples were immediately frozen at -20 °C, freeze-dried, and pulverized (PULVERISETTE 9, Fritsch GmbH, Idar-Oberstein, Germany). Pulverized samples were stored in airtight containers until further analysis. Excreta samples were thawed at 3 °C, weighed, pooled for each hen, and homogenized. Excreta dry matter (DM) was analyzed in triplicate. A subsample of the excreta was freeze-dried, pulverized, and stored as described for digesta. Feed was ground to pass through a 0.5 mm sieve (Ultra Centrifugal Mill ZM 200, Retsch GmbH, Haan, Germany). Samples were analyzed for DM according to the official method in Germany (method no. 3.1) [27]. The pulverized feed, digesta, and excreta samples were analyzed for P, Ca, and titanium by inductively coupled plasma-optical emission spectrometry following wet digestion using a modified method described by Boguhn et al. [28], as detailed by Zeller et al. [29]. Plasma Ca was measured by the Arsenazo method and inorganic P as phosphomolybdate in a Beckman Olympus AU480 photometer by IDEXX BioResearch Vet Med Labor GmbH (Ludwigsburg, Germany). 

The phenotypes of the traits in this study were either direct records during the experiment, derived from obtained samples or were the result of lab analysis of the samples. Calcium content in the excreta (Ca_ex_), Ca retention with (CaR_ex+eggs_) or without (CaR_ex_) considering the excretion via eggs, P content in the excreta (P_ex_), P retention with (PR_ex+eggs_) or without (PR_ex_) considering the excretion via eggs, Ca content in the plasma (Ca_pl_) and in the ileal digesta (Ca_il_), P content in the plasma (P_pl_) and in the ileal digesta (P_il_), InsP_6_ in the ileal digesta (InsP6_il_) as well as MI content in the egg (MI_egg_), in the plasma (MI_pl_), and in the ileal digesta (MI_il_) were the traits of interest. Moreover, phenotypes of body weight (BW) at different phases of the experiment, number of eggs (NoE), and average egg weight (AEW) were regarded as measures of performance, and average daily feed intake (ADFI) as measure of input (see Table 1 for trait descriptions). The latter was also intended as a reference trait to better interpret the results of the focal traits because ADFI is a well-documented trait in quantitative genetic studies with genetic variation in both investigated laying hen strains (see results section). As it is often hard to distinguish whether parts of a broken egg were consumed or not, the number of intact eggs (NoE_int_), the number of defective eggs (NoE_def_), and the number of consumed eggs (NoE_con_), were considered as measure of egg production and of putative egg consumption of the hens. This is a relevant consideration regarding metabolism since egg consumption contributes to Ca and P intake and can therefore compensate for the intended limited nutrient supply. Finally, the egg stage in the oviduct (EW_oviduct_) was considered as an indicator variable of diurnal rhythm. An overview of the phenotypic traits, their abbreviations, the timepoint of phenotyping and a brief description of the procedures to obtain the phenotypes is provided in Table 1.

**Table 1:** Overview of the phenotypic traits assessed in this study

Trait abbreviation	Experimental phase	Trait name and description
BW_1_	start; day 1 of the experiment	Body weight [g] obtained by individual weighing.
BW_15_	start excreta sampling; day 15 of the experiment	Body weight [g] obtained by individual weighing.
BW_18_	end excreta sampling; day 18 of the experiment	Body weight [g] obtained by individual weighing.
BW_SL_	slaughter; day 22, 23 or 24 of the experiment	Body weight [g] obtained by individual weighing.
NoE	start until end of excreta sampling or until slaughter (days 1–18 or days 1–22, 23 or 24)	Number of laid eggs [count] counted. Subscripts indicate the period considered in days, e.g., NoE_1−18_.
NoE_int_	start until end of excreta sampling or until slaughter (days 1–18 or days 1–22, 23 or 24)	Number of intact eggs [count] counted. Subscripts indicate the period considered in days, e.g., NoE_int, 1−18_.
NoE_con_	start until end of excreta sampling or until slaughter (days 1–18 or days 1–22, 23 or 24)	Number of eggs [count] that were in parts consumed by the hen. The events were visually observed and counted. Subscripts indicate the period considered in days, e.g., NoE_con, 1−18_.
NoE_def_	start until end of excreta sampling or until slaughter (days 1–18 or days 1–22, 23 or 24)	Number of eggs [count] that were defective and potentially in parts consumed by the hen. The events were visually observed and counted. Subscripts indicate the period considered in days, e.g., NoE_def_, _1−18_.
AEW	start until end of excreta sampling (days 1–18)	Average egg weight [g]: The total weight of all eggs counted in this period was divided by the number of eggs.
ADFI	start until end of excreta sampling (days 1–18)	Average daily feed intake [g/day]: The feed consumption was quantified by weighing/back weighing of the feed once within the considered period and divided by the number of days.
Ca_ex_	excreta sampling (days 15–18)	Calcium content in the excreta [g/kg DM^a^]: The 4 daily excreta samples were jointly analyzed, and the calcium content was quantified according to Zeller et al. [29] and subsequently divided by the number of days to obtain an average value.
CaR_ex_	excreta sampling (days 15–18)	Calcium retention [%], calculated as the proportion of calcium intake not recovered in excreta during the 4 days of excreta sampling (days 15-18), i.e. (intake - excretion) / intake x 100.
CaR_ex+eggs_	excreta sampling (days 15–18)	Calcium retention [%], calculated as the proportion of calcium intake not recovered in calcium excretion via excreta and eggs during the 4 days of excreta sampling (days 15–18), i.e. (intake − excretion) / intake × 100.
P_ex_	excreta sampling (days 15–18)	Phosphorus content in the excreta [g/kg DM^a^]: The 4 daily excreta samples were jointly analyzed, and the phosphorus content was quantified according to Zeller et al. [29] and subsequently divided by the number of days to obtain an average value.
PR_ex_	excreta sampling (days 15–18)	Phosphorus retention [%], calculated as the proportion of phosphorus intake not recovered in excreta during the 4 days of excreta sampling (days 15–18), i.e. (intake - excretion) / intake x 100.
PR_ex+eggs_	excreta sampling (days 15–18)	Phosphorus retention [%], calculated as the proportion of phosphorus intake not recovered in phosphorus excretion via excreta and eggs during the 4 days of excreta sampling (days 15–18), i.e. (intake − excretion) / intake × 100.
EW_oviduct_	slaughter; day 22, 23 or 24	Weight of the egg stage [g] in the oviduct obtained by weighing.
Ca_pl_	slaughter; day 22, 23 or 24	Calcium content in the plasma [mmol/l] quantified by means of the Arsenazo method.
Ca_il_	slaughter; day 22, 23 or 24	Calcium content in the ileal digesta [g/kg DM^a^] quantified according to Zeller et al. [29].
P_pl_	slaughter; day 22, 23 or 24	Inorganic phosphorus content in the plasma [mmol/l] obtained by photometer analysis (Beckmann Olympus AU480).
P_il_	slaughter; day 22, 23 or 24	Total phosphorus content in the ileal digesta [g/kg DM^a^] quantified according to Zeller et al. [29].
MI_egg_	excreta sampling (days 15–18)	*Myo*-inositol content in the egg [µmol/g DM^a^] collected at excreta sampling from day 15 to day 18 of the experiment.
MI_pl_	slaughter; day 22, 23 or 24	*Myo*-inositol content in the plasma [µmol/g DM^a^] obtained at slaughter.
MI_il_	slaughter; day 22, 23 or 24	*Myo*-inositol content in the ileal digesta [µmol/g DM^a^] obtained at slaughter.
InsP6_il_	slaughter; day 22, 23 or 24	InsP_6_ content in the ileal digesta [µmol/g DM^a^] obtained at slaughter.

^a^*DM* dry matter

All animals were genotyped with the Chicken 50 K-CobbCons 15000986 A SNP-chip (Illumina, San Diego, CA) and filtered according to call rate (≥ 0.95), call frequency (≥ 0.95), cluster separation (≥ 0.4), and heterozygosity excess (≥ -0.3 and ≤ 0.03). Only SNPs assigned to genomic positions (chicken genome assembly GRCg6a) of autosomal chromosomes were considered for analyses. As the number of segregating SNPs on chromosome 16 (*n* = 1 SNP in LSL and *n* = 6 SNPs in LB after minor allele frequency (MAF) filtering) was too small for reliable analyses, this chromosome was discarded. Genotypes were phased and sporadically missing genotypes were imputed using Beagle [30, 31]. Minor allele frequency filtering was applied per strain where all SNPs with MAF ≥ 0.03 were kept. This resulted in 15,875 SNPs in the LSL and 22,559 SNPs in the LB population, which were used for quantitative genetic trait analyses. For population genetic analyses, all SNPs that segregated at least in one strain with a MAF ≥ 0.03 were considered and resulted in 26,944 SNPs.

Only animals with genotype and reliable phenotype information were kept for analysis, implausible phenotypic measures were identified and coded as missing values. Consequently, the number of observations varied depending on the investigated trait. After filtering, data of 180 to 200 LSL and 190 to 198 LB hens were available for further analyses.

### Statistical analysis

#### Population genetic analyses

Population genetic analyses were intended to infer the genetic distance between animals and the genetic differentiation between the two strains, having in mind, that these are hybrid strains, prohibiting an inference regarding the respective purebred populations from the estimated parameters. All analyses were conducted by using the framework provided in PLINK1.9 software [32]. Thereby, both approaches, principal component analysis (PCA) and $\:{F}_{ST}$ value calculation, were applied to the pooled LSL and LB genotype data, according to Jolliffe [33] and Weir and Cockerham [34]. Initial PCA including 20 PCs showed that eigenvalues dropped after the 1st PC and the variance explained by the 2nd PC was below 1%. Consequently, the final analysis considered two PCs. To visualize the genetic distance between animals, the PCA results were plotted based on the resulting eigenvectors. Genetic differentiation between the strains was investigated by means of $\:{F}_{ST}$ value calculation. The average $\:{F}_{ST}$ value across all SNPs that were segregating in at least one of the strains was used for this purpose.

#### Descriptive statistics and phenotypic differences between strains

Descriptive statistics were calculated for all traits in each strain to give an overview of the data. For each trait, differences in the phenotypic variance between strains were assessed by means of Levene’s tests. When the resulting p-value was *p* ≤ 0.05, variance heterogeneity between the strains was confirmed. Differences in phenotypic trait means were tested via Student t-test and Welch t-test for traits with homogeneous and heterogeneous variances, respectively. P-values of *p* ≤ 0.05 indicated significant differences between the strains.

#### Variance component estimation and phenotypic correlations

Given the results of the population genetic analyses and significant phenotypic differences between the two lines, variance component estimation for all traits of interest was conducted separately for each line. The observed distribution of phenotypes deviated significantly from normal distribution (through visual inspection of raw data histograms and Q-Q plots plus Shapiro–Wilk test, *p* < 0.05) for the analyzed traits. To better meet the model assumptions, the phenotypes of each trait were Box-Cox transformed with a specific lambda prior to analyses, using likelihood estimation with grid search implemented in the R package MASS [35], according to Box and Cox [36] to approximate the standard normal distribution:

$$\:f\left(\mathbf{y}\right)=\left\{\begin{array}{c}\frac{{\mathbf{y}}^{\lambda\:}-1}{\lambda\:}\:(\lambda\:\ne\:0)\\\:\mathrm{log}\left(\mathbf{y}\right)\:(\lambda\:=0)\end{array}\right.$$

where $\mathbf{y}$ is the vector of the respective phenotype to be transformed and $\lambda$ is the trait specific transformation parameter, which ranged from − 0.76 to 8.48 and from − 0.46 to 6.57 for the traits recorded in LSL and LB as presented in Additional file 1: Table S3. Subsequently, the Box-Cox transformed phenotypes were z-standardized to enable comparability across traits.

As traits measured in living animals during the experiment and traits measured at slaughter were influenced by different effects, two different models were applied depending on the timepoint of phenotyping. In R (Version 4.2.3; [37]) using ASReml-R (Version 4.1; [38]), the following univariate mixed linear animal model was applied for the traits recorded during the experiment:

1 $$\:\mathbf{y}=\mathbf{X}\mathbf{b}+{\mathbf{Z}}_{a}\mathbf{a}+\mathbf{e}$$

Thereby, $\mathbf{y}$ is the vector of Box-Cox transformed, scaled, and centered (mean = 0, SD = 1) phenotypes, $\mathbf{b}$ is the vector of fixed effects, $\mathbf{a}$ is the vector of random animal effects, modeled as $\:\mathbf{a}\sim {N}(0,\mathbf{G}{\sigma\:}_{a}^{2})$ with $\:{\sigma\:}_{a}^{2}$ being the additive genetic variance and $\:\mathbf{G}$ the additive genomic relationship matrix according to the first method described by VanRaden [39], as $\:\mathbf{G}=\frac{\mathbf{Z}{\mathbf{Z}}^{\mathrm{T}}}{\sum_{j}2{p}_{j}(1-{p}_{j})}$, where $\:\mathbf{Z}$ is an $\:n\times\:m$ matrix of centred genotypes with $\:n$ the number of animals and $\:m$ the number of SNPs, and $\:{p}_{j}$ is the allele frequency of the reference allele at SNP $\:j$. **X** denotes the design matrix for the fixed effects to the respective observation and $\:{\mathbf{Z}}_{a}$ the incidence matrix for the random effects. Vector $\:\mathbf{e}$ contains the model residuals, assumed to follow a normal distribution as $\:\mathbf{e}\sim{N}(0,\mathbf{I}{\sigma\:}_{e}^{2})$, with $\:{\sigma\:}_{e}^{2}$ being the residual variance and $\:\mathbf{I}$ the identity matrix.  

For the traits recorded at slaughter, a random effect of the slaughter day ($\:\mathbf{s}\mathbf{l}\mathbf{d}$) was added, which resulted in:

2 $$\:\mathbf{y}=\mathbf{X}\mathbf{b}+{\mathbf{Z}}_{a}\mathbf{a}+\mathbf{W}\mathbf{s}\mathbf{l}\mathbf{d}+\mathbf{e}$$

where $\:\mathbf{s}\mathbf{l}\mathbf{d}$ is modeled as $\:\mathbf{s}\mathbf{l}\mathbf{d}\sim {N}(0,\:\mathbf{I}{\sigma\:}_{sld}^{2})$, with slaughter day variance $\:{\sigma\:}_{sld}^{2}$, and the corresponding incidence matrix $\:\mathbf{W}$. All other terms are defined in Eq. (1).

Fixed effects were tested for significance (*p* ≤ 0.05) by the application of conditional Wald tests. Factors with significant systematic effect on at least one of the traits were kept in the model. For Eq. (1), these were the effects of the cohort and the metabolic unit. For Eq. (2), the cohort effect and the effect of the egg stage in the oviduct at slaughter were included as fixed effects. Measures of egg performance (NoE, AEW, NoE_int_, NoE_con_, NoE_def_) were fitted in both models. The measurements of NoE_int_, NoE_con_, and NoE_def_ were fitted as linear and quadratic regression variables to correct for the potential and definitive intake of egg components. Significance of fixed effects are shown in Additional file 1: Tables S4 and S5. In Eq. (1), the various egg measurements were counted from the start of the experiment until the end of excreta sampling, whereas in Eq. (2) they were counted until the day of slaughter. The Bayesian information criterion was considered to assess the quality of the model fit [40].

Heritability ($\:{h}^{2}$) of the traits recorded during the experiment was calculated as $\:{h}^{2}=\frac{{\sigma\:}_{a}^{2}}{{\sigma\:}_{a}^{2}+{\sigma\:}_{e}^{2}}$ using Eq. (1) and $\:{h}^{2}=\frac{{\sigma\:}_{a}^{2}}{{\sigma\:}_{a}^{2}+{\sigma\:}_{sld}^{2}+{\sigma\:}_{e}^{2}}$ for the traits recorded at slaughter using Eq. (2). The significance of the heritability estimates was assessed by likelihood-ratio tests, testing whether the additive genetic variance differs significantly from zero.

Finally, Pearson phenotypic correlations were calculated separately for the pre-corrected traits for LSL and LB. The phenotypes were pre-corrected using a mixed linear model accounting for the fixed effects of Eq. (1) for the measurements on live animals during the experiment and the traits measured at slaughter were pre-corrected for the fixed effects plus the random slaughter day effect according to Eq. (2). Note that BW measurements were solely used to show differences between the strains at a phenotypic level and for variance component estimation but were not used to calculate phenotypic trait correlations.

## Results

### Population genetic analyses

**Fig. 2:**
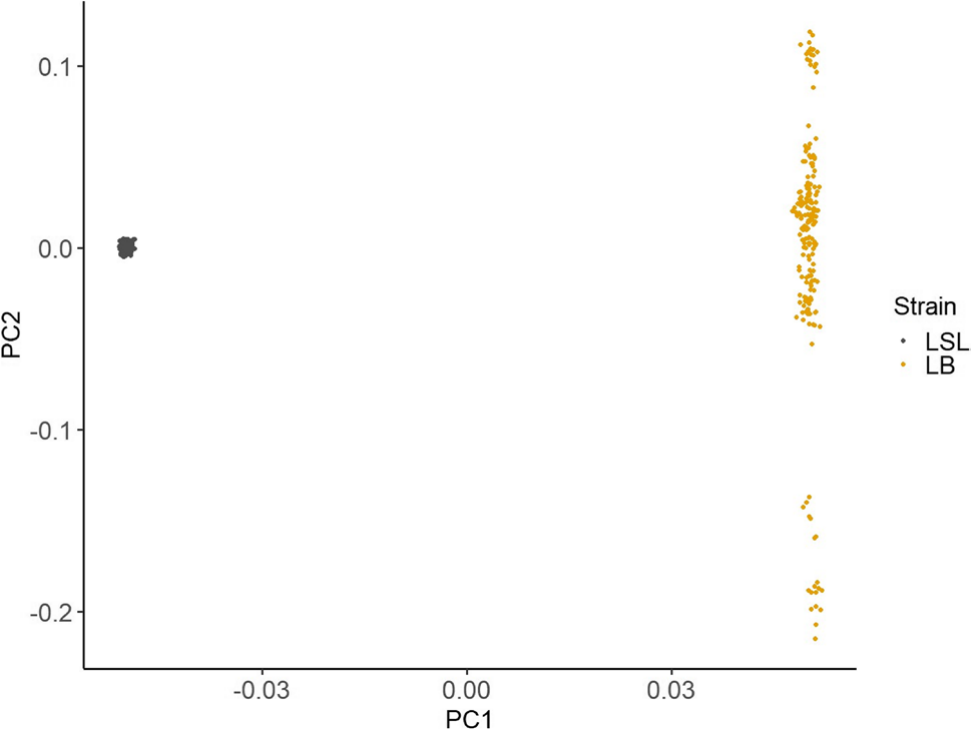
Genetic distance within and between Lohmann Selected Leghorn (LSL) and Lohmann Brown (LB) strain based on 50K SNP-chip information

Population genetic analyses revealed a large genetic differentiation between the two strains, which is visualized in Fig. 2 and supported by an average $\:{F}_{ST}$ value of $\:{F}_{ST}$ = 0.26. Within strains, PCA plot (Fig. 2) showed different patterns for LSL and LB. Genetic distances between LSL hens seemed to be much smaller compared with the genetic distances between LB hens, indicating putatively lower genetic diversity in LSL. Similarly, the number of SNPs that segregated in LB was 1.42 times as large as the number of SNPs that segregated in LSL, and only 11,490 SNPs segregated in both strains with MAF ≥ 0.03.

### P and Ca metabolism and performance in the LSL and LB strain

Eighteen out of 25 traits had significantly different phenotypic means and almost half of the traits differed significantly in variance (Table 2). Phenotypic variability was observed for all traits and in both strains, the SD values imply larger phenotypic variation in LB for most of the traits. Regarding the focused traits of P and Ca metabolism, significant differences were observed between strains for most of the traits. Whereas Ca content measured in excreta, plasma, or ileal digesta did not differ between strains, average Ca retention was significantly higher in LSL (CaR_ex_ + 5.17%, CaR_ex+eggs_ + 31.45%). The values of these traits varied widely in both strains, including negative values. Similarly, a wide range of P retention (including negative values) was observed with significant differences between the strains. Lohman Selected Leghorn hens had a significantly higher phenotypic mean regarding PR_ex_ (+ 7.90%), although they excreted significantly more P via excreta. For the trait PR_ex+eggs_, the higher trait mean of LSL was not significant; however, the trait variances were heterogeneous across strains. Plasma P content was significantly higher in LB than in LSL; such differences were not observed in the ileal digesta. *Myo*-inositol traits and InsP6_il_ differed significantly between the strains. Thereby, LB had on average higher concentrations of MI measured in the egg and ileal digesta but lower concentrations of MI_pl_ and InsP6_il_. Lohmann Brown hens were significantly heavier and produced a slightly, but significantly, lower number of eggs than LSL hens (Table 2). Trait mean and variance differences were present for NoE_int_, NoE_def_ and NoE_con_, whereby LB had significantly more defective and consumed eggs. EW_oviduct_ was on average higher in LB compared with LSL. No notable differences were observed for AEW, but variance differences were observed for ADFI. Significant systematic effects responsible for trait mean differences are presented in Additional file 1: Tables S4 and S5, revealing a significant impact of the cohort and unit on most traits in both strains. Effects of EW_oviduct_ as well as the measures of intact, broken, or consumed eggs were significant for several traits of the P and Ca metabolism and were more pronounced in the LB strain. 

**Table 2:** Descriptive statistics of the analyzed traits in Lohmann Selected Leghorn (LSL) and Lohmann Brown (LB) and phenotypic differences between the strains

Trait^a^		LSL					LB						
	Unit	*n*	Mean	SD	Min	Max	*n*	Mean	SD	Min	Max	*p*_Levene’s_	*p*_t−test_
BW_1_	g	200	1,715.49	135.34	1,193.00	2,035.00	198	1,935.47	144.40	1,570.00	2,394.00	0.51	< 0.01*
BW_15_	g	200	1,662.02	116.26	1,344.00	1,963.00	198	1,861.67	142.54	1,422.00	2,203.00	0.02*	< 0.01*
BW_18_	g	200	1,664.39	122.48	1,371.00	1,986.00	198	1,869.12	152.81	1,390.00	2,243.00	< 0.01*	< 0.01*
BW_SL_	g	200	1,688.27	127.86	1,318.00	2,012.00	198	1,907.19	153.94	1,467.00	2,294.00	0.01*	< 0.01*
NoE_1−18_	count	200	16.23	1.55	5.00	18.00	198	15.78	1.97	7.00	18.00	0.01*	0.01*
NoE_int, 1−18_	count	200	15.8	1.87	5.00	18.00	198	13.99	4.22	1.00	18.00	< 0.01*	< 0.01*
NoE_con, 1−18_	count	200	0.08	0.34	0.00	3.00	198	0.47	1.43	0.00	11.00	< 0.01*	< 0.01*
NoE_dam, 1−18_	count	200	0.27	0.71	0.00	5.00	198	1.18	2.35	0.00	10.00	< 0.01*	< 0.01*
AEW	g	200	61.88	4.02	50.00	73.00	198	62.07	4.14	51.00	75.00	0.85	0.65
ADFI	g/d	200	102.62	11.43	66.00	131.00	198	100.06	15.27	35.00	134.00	0.01*	0.06
Ca_ex_	g/kg DM^b^	200	44.63	10.38	20.50	81.80	198	46.06	11.37	22.60	90.40	0.18	0.19
CaR_ex_	%	200	67.08	10.46	8.00	86.00	198	63.78	11.25	6.00	83.00	0.12	< 0.01*
CaR_ex+eggs_	%	200	11.37	11.34	-31.00	37.00	198	8.65	13.87	-48.00	40.00	0.01*	0.03*
P_ex_	g/kg DM^b^	200	9.72	1.06	7.50	13.90	198	9.42	1.07	6.90	12.20	0.47	< 0.01*
PR_ex_	%	200	30.86	9.71	-1.00	54.00	198	28.60	10.29	-28.00	48.00	0.52	0.03*
PR_ex+eggs_	%	200	2.90	11.45	-37.00	27.00	198	0.88	13.21	-73.00	32.00	0.20	0.10
EW_oviduct_	g	187	44.67	11.63	0.00	66.00	190	51.45	10.13	28.00	87.00	0.16	< 0.01*
Ca_pl_	mmol/l	198	6.95	1.31	3.80	12.60	197	7.12	1.00	4.20	10.30	< 0.01*	0.13
Ca_il_	g/kg DM^b^	188	44.87	30.39	8.90	250.50	197	43.51	34.31	9.10	224.00	0.82	0.68
P_pl_	mmol/l	180	1.06	0.34	0.40	1.80	190	1.21	0.30	0.50	2.40	0.02*	< 0.01*
P_il_	g/kg DM	188	9.90	3.33	2.40	41.30	197	10.08	2.61	2.80	19.50	0.92	0.55
MI_egg_	µmol/g DM^b^	200	2.53	0.33	1.80	3.40	190	2.46	0.35	1.70	3.50	0.47	0.03*
MI_pl_	µmol/g DM^b^	200	0.14	0.04	0.04	0.26	198	0.16	0.04	0.07	0.42	0.67	< 0.01*
MI_il_	µmol/g DM^b^	194	2.13	1.37	0.30	10.40	197	3.95	2.62	0.70	18.70	< 0.01*	< 0.01*
InsP6_il_	µmol/g DM^b^	194	30.01	8.76	4.60	54.20	198	32.20	9.86	0.50	63.70	0.11	0.02*

The sample size (n), phenotypic mean (Mean), its standard deviation (SD), minimum (Min) and maximum value (Max) are given. Significant differences of trait variance (Levene’s test) or trait mean (t-test, Welch-corrected for traits with heterogeneous variances across strains) between strains were assessed, the resulting p-values (*p*_Levene’s_ and *p*_t−test_) are given. ^a^Full trait descriptions are given in Table 1; ^b^*DM* dry matter; *p-values of *p* ≤ 0.05 indicate statistical differences

### Variance components and phenotypic correlations

The models did converge for all traits in both strains; however, estimates of additive genetic variance were only obtained for 13 and 17 traits for LSL and LB, respectively (Table 3). The slaughter day effect did not, or only marginally, contribute to the phenotypic variance of the traits recorded at slaughter. Regarding the Ca or P measurements, only Ca_pl_ showed a significant additive genetic variance and moderate heritability ($\:h^2$ = 0.27) in the LB strain. Among the other traits of metabolism, MI_egg_ and MI_pl_ showed a significant and substantial additive genetic component in both strains ($\:h^2$ of 0.69 and 0.43 for LSL and 0.55 and 0.36 for LB, respectively), and MI_il_ in the LB strain ($\:h^2$ = 0.60). The heritabilities of BW ranged from 0.66 to 0.83 for LSL and from 0.84 to 0.88 for LB, depending on the respective observation time point. Moderately high and significant heritability estimates were observed for ADFI in both strains. The AEW exhibited a high heritability ($\:h^2$ = 0.70) in the LB strain, whereas genetic variation for this trait was minimal in the LSL strain. Although the standard errors were large due to the limited sample size, the estimates of additive genetic variance for some traits were sufficiently large to yield statistically significant results.

**Table 3:** Results of the variance component estimation for the Lohmann Selected Leghorn (LSL) and Lohmann Brown (LB) strain presented for all traits with an estimable additive genetic variance component

Strain	Trait^a^	h^2^ (SE)	*p*-value^b^	V_A_ (SE)	V_SLD_ (SE)	V_E_ (SE)
LSL	BW_1_	0.66 (0.16)*	< 0.01	0.73 (0.25)	n.m.^c^	0.38 (0.12)
	BW_15_	0.82 (0.12)*	< 0.01	0.95 (0.25)	n.m.^c^	0.21 (0.10)
	BW_18_	0.83 (0.12)*	< 0.01	1.03 (0.27)	n.m.^c^	0.21 (0.11)
	BW_SL_	0.78 (0.13)*	< 0.01	0.97 (0.27)	n.m.^c^	0.27 (0.11)
	AEW	0.10 (0.16)	0.62	0.06 (0.10)	n.m.^c^	0.52 (0.12)
	ADFI	0.66 (0.18)*	< 0.01	0.71 (0.27)	n.m.^c^	0.37 (0.13)
	Ca_ex_	0.22 (0.18)	0.07	0.20 (0.16)	n.m.^c^	0.69 (0.12)
	CaR_ex_	0.17 (0.16)	0.16	0.14 (0.14)	n.m.^c^	0.69 (0.11)
	CaR_ex+eggs_	0.03 (0.10)	0.74	0.02 (0.08)	n.m.^c^	0.75 (0.10)
	Ca_il_	0.03 (0.12)	0.41	0.02 (0.11)	n.e.^d^	0.90 (0.13)
	P_il_	0.07 (0.13)	0.25	0.03 (0.06)	n.e.^d^	0.45 (0.06)
	MI_egg_	0.69 (0.18)*	0.02	0.88 (0.32)	n.m.^c^	0.39 (0.15)
	MI_pl_	0.43 (0.21)*	< 0.01	0.47 (0.21)	0.03 (0.04)	0.59 (0.14)
LB	BW_1_	0.88 (0.11)*	< 0.01	1.08 (0.27)	n.m.^c^	0.14 (0.10)
	BW_15_	0.84 (0.12)*	< 0.01	0.94 (0.25)	n.m.^c^	0.17 (0.10)
	BW_18_	0.86 (0.12)*	< 0.01	0.93 (0.24)	n.m.^c^	0.15 (0.10)
	BW_SL_	0.85 (0.12)*	< 0.01	0.95 (0.25)	n.m.^c^	0.16 (0.10)
	AEW	0.70 (0.15)*	< 0.01	0.74 (0.23)	n.m.^c^	0.32 (0.10)
	ADFI	0.53 (0.19)*	< 0.01	0.42 (0.18)	n.m.^c^	0.38 (0.10)
	Ca_ex_	0.23 (0.18)	0.06	0.21 (0.16)	n.m.^c^	0.68 (0.12)
	CaR_ex_	0.16 (0.16)	0.20	0.12 (0.13)	n.m.^c^	0.67 (0.10)
	CaR_ex+eggs_	0.03 (0.12)	0.78	0.02 (0.08)	n.m.^c^	0.66 (0.09)
	P_ex_	0.15 (0.16)	0.28	0.09 (0.09)	n.m.^c^	0.49 (0.08)
	Ca_pl_	0.27 (0.17)*	0.01	0.8 (0.18)	n.e.^d^	0.77 (0.13)
	Ca_il_	0.03 (0.10)	0.38	0.02 (0.09)	0.12 (0.09)	0.77 (0.11)
	P_pl_	0.11 (0.14)	0.14	0.11 (0.13)	0.05 (0.06)	0.85 (0.13)
	MI_egg_	0.55 (0.16)*	< 0.01	0.51 (0.18)	n.m.^c^	0.41 (0.10)
	MI_pl_	0.36 (0.20)*	0.01	0.35 (0.21)	0.06 (0.06)	0.58 (0.13)
	MI_il_	0.60 (0.19)*	< 0.01	0.61 (0.24)	n.e.^d^	0.41 (0.12)
	InsP6_il_	0.12 (0.16)	0.19	0.07 (0.10)	0.02 (0.03)	0.51 (0.08)

The heritability ($\:h^2$), its p-value, additive genetic variance (V_A_), slaughter day variance (V_SLD_, only modeled for traits measured at slaughter) and residual variance (V_E_) are presented. Standard errors of the estimates (SE) are given in parentheses. ^a^Full trait descriptions are given in Table 1; ^b^obtained by likelihood ratio test; ^c^not modeled; ^d^not estimable; *significant (*p* ≤ 0.05) heritabilities

The results of the pre-corrected phenotypic correlations are shown in Table 4. It is noticeable that there were strong significant correlations (*p* ≤ 0.05) between InsP6_il_ and Ca_il_ and P_il_, between P_il_ and Ca_il_, between P_pl_ and Ca_pl_, and between PR_ex+eggs_ and CaR_ex+eggs_ for both strains.

**Table 4:** Pearson phenotypic correlations of pre-corrected phenotypes for Lohmann Selected Leghorn (LSL) below the diagonal and Lohmann Brown (LB) above the diagonal

NoE	NoE	AEW	ADFI	Ca_ex_	CaR_ex_	CaR_ex+eggs_	*P*_ex_	PR_ex_	PR_ex+eggs_	Ca_pl_	Ca_il_	*P*_pl_	*P*_il_	MI_egg_	MI_pl_	MI_il_	InsP6_il_
		0.07	0.19*	0.06	-0.04	-0.16*	0.02	-0.01	-0.08	0.06	0.01	0.10	-0.06	-0.04	0.00	-0.05	-0.07
AEW	0.05		0.49*	0.12	-0.11	-0.09	0.03	-0.03	-0.02	-0.09	-0.05	0.02	-0.21*	0.11	-0.05	0.13	-0.24*
ADFI	0.01	0.50*		0.27*	-0.23*	0.10	-0.09	0.02	0.19*	-0.13	0.15*	0.06	-0.13	0.05	-0.04	0.00	-0.11
Ca_ex_	0.07	-0.01	0.18*		-0.92*	-0.54*	-0.40*	0.15*	0.24*	0.01	0.14*	0.15*	-0.04	-0.17*	-0.20*	-0.11	-0.07
CaR_ex_	-0.18*	0.04	-0.19*	-0.93*		0.62*	0.43*	0.13	0.00	0.05	-0.21*	-0.15*	0.11	0.14	0.14*	0.11	0.12
CaR_ex+eggs_	0.02	0.03	0.07	-0.71*	0.74*		0.24*	0.23*	0.48*	0.04	-0.09	-0.03	-0.01	0.13	0.11	0.12	-0.01
P_ex_	-0.05	0.01	-0.19*	-0.42*	0.44*	0.16*		-0.50*	-0.47*	0.14	-0.03	0.14	0.03	-0.05	0.06	-0.04	0.03
PR_ex_	-0.12	0.04	-0.01	-0.05	0.30*	0.38*	-0.44*		0.89*	0.06	-0.16*	-0.14	0.12	-0.01	-0.10	0.12	0.08
PR_ex+eggs_	0.02	0.04	0.15*	0.05	0.14	0.52*	-0.51*	0.90*		0.04	-0.08	-0.07	0.04	-0.01	-0.08	0.11	0.00
Ca_pl_	-0.17*	0.04	-0.13	0.01	-0.02	-0.11	-0.09	0.04	-0.03		0.01	0.40*	0.20*	-0.13	-0.03	-0.12	0.16
Ca_il_	-0.10	0.11	0.08	0.25*	-0.24*	-0.17*	-0.06	-0.07	-0.03	0.15*		0.06	-0.41*	-0.02	0.09	-0.24*	-0.33*
P_pl_	-0.11	0.08	-0.01	0.02	-0.03	-0.10	-0.04	-0.02	-0.06	0.53*	-0.05		-0.11	-0.07	0.11	-0.11	-0.09
P_il_	-0.01	-0.03	-0.06	-0.08	0.09	0.09	0.11	0.01	0.02	0.01	-0.33*	-0.07		-0.10	-0.04	0.04	0.94*
MI_egg_	0.03	0.20*	0.11	-0.23*	0.22*	0.25*	0.04	0.05	0.08	-0.12	0.00	0.01	0.11		0.14	0.00	-0.05
MI_pl_	-0.09	0.09	-0.12	-0.21*	0.26*	0.21*	0.08	0.11	0.09	0.15*	0.01	0.30*	0.14	0.25*		-0.05	0.00
MI_il_	0.02	0.10	-0.03	-0.10	0.09	0.08	-0.01	0.02	0.02	0.02	-0.12	0.01	0.05	0.03	0.10		-0.07
InsP6_il_	-0.04	0.03	-0.02	-0.10	0.10	0.12	0.11	-0.01	0.01	0.06	-0.24*	-0.01	0.92*	0.16*	0.19*	0.05	

Full trait descriptions are given in Table 1; *correlations with a p-value of *p* ≤ 0.05 indicate statistical significance

## Discussion

### Experimental design

The strains were hybrids, which has to be considered in the interpretation of the estimated genetic parameters [13] and the population differentiation $\:{F}_{ST}$ parameter, which are usually applied in segregating purebred populations.

The animal experiment was carried out with a total of 400 laying hens, 200 LSL and 200 LB hens each, which were kept individually in metabolic units and phenotyped for numerous traits related to P and Ca metabolism. To date, only a few studies have investigated the genetic basis of P and Ca metabolism in poultry. Most of the studies conducted were carried out on non-laying animals (e.g., [13]). Our experiment on digestive processes and metabolism during the egg-laying phase is therefore unique, particularly in terms of sample size. Both the LSL and LB hens were raised in the same environment and data were completely generated under similar conditions since hens of both strains were evenly distributed across experimental periods. The latter allowed for a reliable comparison of the trait heritabilities between the strains.

However, the immense effort required for phenotyping these mainly hard-to-measure traits limited the experimental capacity in terms of sample size, which is usually desired to be larger for quantitative genetic analyses. This came along with four consecutive experimental periods. The fixed effects of the cohorts (1 to 4) were highly significant for almost all traits. The cohorts thereby did not only reflect the runs with observer and handler effects but also served to illustrate the age structure of the animals. As all hens hatched at the same time and the cohorts started with a time difference of four weeks, the hens were four weeks older than those of the previous cohort (Fig. 1). To account for potential age effects and systematic differences between the cohorts this was included in Eqs. (1) and (2) as a fixed cohort effect.

The hens were fed a lower P supply than currently recommended by the GfE [26]. Diets lacked exogenous phytase and mineral P supplements (Additional file 1: Tables S1 and S2) to enable the animals to fully exploit their genetic potential in P and Ca utilization. An aspect that required particular attention was the correct identification and consideration of the eggs laid, destroyed and partially eaten. The intact and indeed (partially) eaten eggs were very well recorded. A modeling of the intact (NoE_int_), potentially eaten (NoE_def_) and eaten eggs (NoE_con_) was done, because they addressed the intake of egg components, as these potentially influence P and Ca metabolism.

### Population genetic differences between LSL and LB

The PCA and the average $\:{F}_{ST}$ value of 0.26 clearly indicate large differentiation between the strains, which is in line with the results of other studies (e.g., [19]). At the genomic and transcriptomic level, Iqbal et al. [41] identified 17 highly divergent candidate genes using $\:{F}_{ST}$-based gene aggregation analysis, also indicating substantial genetic differences between LB and LSL. Heumann-Kiesler et al. [20] investigated the differences between these strains with regard to mitochondrial DNA and showed genetic variation in LB but not in LSL. This is supported by the results of the present study. While PCA revealed that LB hens formed a rather loose cluster along the second principal component (PC2), all LSL hens clustered very closely together in one single cluster. Also, the number of SNPs that segregated in LSL was strongly decreased compared with the other strain. These results imply that genetic diversity in LB seemed to be remarkably larger than in LSL, in which the detectable genetic diversity was negligibly small. These results are based on the genomic information captured by segregating variants. In this study, hens were genotyped with the Chicken 50 K-CobbCons 15000986 A SNP-chip (Illumina, San Diego, CA). Such genotyping tools are designed to be representative for a maximal number of chicken populations and breeds; however, they may not necessarily represent hybrids of high yielding founder populations in the best way. The loss of SNPs due to MAF filters observed during SNP data processing may indicate that this could have impacted the presented results, particularly for LSL where this was more pronounced. The population genetic analyses were primarily intended to provide a better and more complete explanation and interpretation of the two studied populations rather than a general comparison of different laying hen strains, which would have required the consideration of genetic origins beyond LSL and LB.

### P and Ca metabolism and strain differences at the phenotypic level

Significant phenotypic differences were found between LSL and LB for the majority of P and Ca metabolism traits (see Table 2), showing that the physiology of P and Ca metabolism was, to a notable extent, strain specific. It underlines the physiological differences between these strains with regard to minerals found in the pre-laying period [18], laying period [23] or gastrointestinal phytate degradation [3, 11] as well as in immunity [42], bone metabolism [43, 44], and behavior [45] between LSL and LB.

The number of intact and putatively consumed eggs had a significant influence on traits related to P metabolism, in tendency being more pronounced in the LB strain (Additional file 1: Tables S4 and S5). Hens of the LB strain had fewer intact eggs and consumed significantly more egg material than LSL hens (Table 2). It is known that LB and other brown layer genetics differ significantly from LSL and other white layer genetics in terms of laying behavior by showing significantly lower nest acceptance as well as differences in pecked and partly consumed eggs [46, 47]. With regard to possible differences in egg shell stability between LSL and LB, which could explain the greater number of broken eggs, Habig and Distl [48] were unable to find any significant differences and Habig et al. [49] found better eggshell stability for LB hens. Note that these animals in their study were not restricted for P like in the current study. Our findings suggest that the two strains either could have different strategies to cope with limiting mineral supply or do have different mineral demands, have metabolic stress or attempt to cope with the lack of stimulation and the unenriched environment in the metabolic units. Habig et al. [49] showed for example, that LSL had an increased Ca requirement due to higher egg weights and eggshell weights as well as higher bone strength compared to LB.

Besides insights into the differences between strains, the study provided a very large database for investigating P and Ca metabolism in laying hens per se compared with most physiological studies. Thereby, the variety of traits measured in the same individuals enabled to draw a comprehensive picture about relationships between traits (Table 4) as well as the identification of systematic effects that impact the traits (Additional file 1: Tables S4 and S5). The significant and high correlations between the P and Ca traits in the ileum, excreta and plasma in both strains exemplify the strong interrelationships between those traits in the laying hen with its unique P and Ca metabolism. The laying hen has a varying need for Ca for the eggshell calcification throughout the day and the provision of Ca that occurs via feed and mobilization of the medullary bone in times of insufficient Ca intake with the feed. As the bone’s inorganic matrix consists mainly of hydroxyapatite, P is also mobilized each time Ca is needed for egg production [50] which can be seen in the present correlations. The positive relationship between InsP6_il_ and P_il_ confirms that this mineral has a detrimental effect on the degradation and utilization of InsP_6_-P. It shows once more that dietary provision of this mineral should be kept on a low level to allow for an optimal InsP_6_-P usage [11, 51].

Due to the limited sample size, no genetic correlations were estimated. Larger sample sizes will be required in the future to evaluate these parameters, which are important for understanding the genetic relationship between traits.

### P and Ca metabolism and strain differences at the quantitative genetic level

Heritable variation was generally more pronounced in the LB strain, which is consistent with the larger genetic diversity of LB observed in the population genetic analysis. This indicates that the genetic background of the strains was a relevant driver of the observed trait differences. Additive genetic variance was obviously too small to estimate in some of the investigated traits and the study could only prove significant heritable variation for 3 and 6 traits in the LSL and LB strain, respectively (see Table 3). Thereof, 2 in LSL and 4 in LB were traits of P and Ca metabolism. Differences in mineral metabolism between these strains were also reported at the genetic level [49]. These authors suggested the possibility that LSL hens may have downregulated their P metabolism to meet the enormous Ca requirement, which is reflected in reduced P absorption in the intestine and thus a constant P plasma content in the blood. In addition, no additive genetic variance of P traits was found for the LSL animals, whereas additive genetic variances for P_pl_ and P_ex_ were detected in the LB hens.

In contrast to the present results, other studies estimated significant additive genetic variance for P and Ca utilization in a setting of growing Japanese quail [13] and for phytate P bioavailability in broilers and layers [14, 15]. Worth noting is that only genetic variance explained by the SNPs in the marker panel can be captured. In the present data, the loss of SNPs due to MAF filtering might have compromised the ability to capture the full additive genetic variance. Statistical inferences were additionally impaired by sample size, which was relatively small for quantitative genetic analyses and led to high standard errors. It should also be mentioned that LSL and LB are hybrid genotypes, whereby the genetic interpretation of these crossbreds differs from that of purebreds. In addition, non-additive effects were not explicitly modeled due to the limited sample size, although they may contribute to genetic variance, particularly in hybrid populations.

## Conclusion

This study provided a comprehensive insight into traits referring to P and Ca metabolism under standardized conditions in the two commercial laying hen strains LSL and LB in the peak of egg laying. This unique setting revealed important findings about P and Ca physiology, which differed between the strains at the phenotypic and quantitative genetic level. The hens’ genetics appeared to be a relevant driver of P and Ca metabolism, with LB showing more variability. The study also confirmed population genetic differentiation between the strains and revealed larger genetic diversity in LB compared with LSL when using 50K SNP chip genotypes. Despite the detected strain differences, correlations among the traits of P and Ca metabolism indicate that the general relationships between traits are comparable in both strains. Although the complexity of the data structure was high, the study provides important insights derived from its novel and complex phenotypic traits and opens up new perspectives for understanding the genetic basis of P and Ca metabolism. These pathways are relevant to the animals’ physiological condition in laying hens and putatively impact health-related traits, however, this requires further research. 

## Supplementary Information

Supplementary Material 1: Additional file 1: Table S1. Ingredients and calculated composition of the experimental diet, Table S2. Analyzed composition of the experimental diet, Table S2. Analyzed composition of the experimental diet, Table S3. Box-Cox transformation lambda values of the analyzed traits in Lohmann Selected Leghorn (LSL) and Lohmann Brown (LB), Table S4. Fixed model effects for each trait in the Lohmann Selected Leghorn (LSL) strain, Table S5. Fixed model effects for each trait in the Lohmann Brown (LB) strain.

## Data Availability

The datasets used and/or analyzed during the current study are available upon reasonable request to the corresponding author.

## References

[1] Rodehutscord M. Chapter 10 Interactions between minerals and phytate degradation in poultry – challenges for phosphorus digestibility assays. In: Rodehutscord M, Walk CL, Kühn I, Stein HH, Kidd MT, editors. Phytate destruction: Consequences for precision animal nutrition. Wageningen: Wageningen Academic; 2016. pp. 167–78.

[2] Selle PH, Cowieson AJ, Ravindran V. Consequences of calcium interactions with phytate and phytase for poultry and pigs. Livest Sci. 2009;124:126–41.

[3] Sommerfeld V, Huber K, Bennewitz J, Camarinha-Silva A, Hasselmann M, Ponsuksili S, et al. Phytate degradation, myo-inositol release, and utilization of phosphorus and calcium by two strains of laying hens in five production periods. Poult Sci. 2020;99:6797–808. 33248595 10.1016/j.psj.2020.08.064 PMC7704748

[4] Hanauska A, Sommerfeld V, Schollenberger M, Huber K, Rodehutscord M. Endogenous mucosal phosphatases characterization in duodenum brush border membrane of laying hens. Front Physiol. 2025;16:1581088. 40241716 10.3389/fphys.2025.1581088 PMC11999836

[5] Rodehutscord M, Sommerfeld V, Kühn I, Bedford MR, Phytases. Potential and Limits of Phytate Destruction in the Digestive Tract of Pigs and Poultry. In: Bedford MR, Partridge G, Walk CL, Hruby M, Evans C, Irving H, et al. editors. Enzymes in Farm Animal Nutrition. GB: CABI; 2022. pp. 124–52.

[6] Omotoso AO, Reyer H, Oster M, Ponsuksili S, Wimmers K. Jejunal microbiota of broilers fed varying levels of mineral phosphorus. Poult Sci. 2023;102:103096. 37797492 10.1016/j.psj.2023.103096 PMC10562922

[7] Huber K. Cellular myo-inositol metabolism. In: Rodehutscord M, Walk CL, Kühn I, Stein HH, Kidd MT, editors. Phytate destruction: Consequences for precision animal nutrition. Wageningen: Wageningen Academic; 2016. pp. 53–60.

[8] Gonzalez-Uarquin F, Rodehutscord M, Huber K. Myo-inositol: its metabolism and potential implications for poultry nutrition-a review. Poult Sci. 2020;99:893–905. 32036985 10.1016/j.psj.2019.10.014 PMC7587644

[9] Sommerfeld V, Hanauska A, Huber K, Bennewitz J, Camarinha-Silva A, Feger M, et al. Effects of myo-inositol supplementation in the diet on myo-inositol concentrations in the intestine, blood, eggs, and excreta of laying hens. Poult Sci. 2025;104:104545. 39579515 10.1016/j.psj.2024.104545 PMC11617940

[10] Marounek M, Skřivan M, Rosero O, Rop O. Intestinal and total tract phytate digestibility and phytase activity in the digestive tract of hens fed a wheat-maize-soyabean diet. J Anim Feed Sci. 2010;19:430–9.

[11] Sommerfeld V, Omotoso AO, Oster M, Reyer H, Camarinha-Silva A, Hasselmann M, et al. Phytate degradation, transcellular mineral transporters, and mineral utilization by two strains of laying hens as affected by dietary phosphorus and calcium. Anim (Basel). 2020;10:1736. 10.3390/ani10101736 PMC7598718 32987788

[12] Marounek M, Skřivan M, Dlouhá G, Břeňová N. Availability of phytate phosphorus and endogenous phytase activity in the digestive tract of laying hens 20 and 47 weeks old. Anim Feed Sci Technol. 2008;146:353–9.

[13] Beck P, Piepho H-P, Rodehutscord M, Bennewitz J. Inferring relationships between phosphorus utilization, feed per gain, and bodyweight gain in an F2 cross of Japanese quail using recursive models. Poult Sci. 2016;95:764–73. 26740136 10.3382/ps/pev376

[14] Zhang W, Aggrey SE, Pesti GM, Edwards HM, Bakalli RI. Genetics of phytate phosphorus bioavailability: heritability and genetic correlations with growth and feed utilization traits in a randombred chicken population. Poult Sci. 2003;82:1075–9. 12872962 10.1093/ps/82.7.1075

[15] Ankra-Badu GA, Pesti GM, Aggrey SE. Genetic interrelationships among phosphorus, nitrogen, calcium, and energy bioavailability in a growing chicken population. Poult Sci. 2010;89:2351–5. 20952697 10.3382/ps.2010-00870

[16] Vollmar S, Haas V, Schmid M, Preuß S, Joshi R, Rodehutscord M, Bennewitz J. Mapping genes for phosphorus utilization and correlated traits using a 4k SNP linkage map in Japanese quail (Coturnix japonica). Anim Genet. 2021;52:90–8. 33140443 10.1111/age.13018

[17] Fawcett DL, Casey-Trott TM, Jensen L, Caston LJ, Widowski TM. Strain differences and effects of different stocking densities during rearing on the musculoskeletal development of pullets. Poult Sci. 2020;99:4153–61. 32867958 10.1016/j.psj.2020.05.046 PMC7598119

[18] Khanal T, Widowski T, Bédécarrats G, Kiarie E. Effects of pre-lay dietary calcium (2.5 vs. 4.0%) and pullet strain (Lohmann Brown vs. Selected Leghorn LSL-Lite) on calcium utilization and femur quality at 1st through to the 50th egg2. Poult Sci. 2019;98:4919–28. 31065713 10.3382/ps/pez245

[19] Brekke C, Groeneveld LF, Meuwissen THE, Sæther N, Weigend S, Berg P. Assessing the genetic diversity conserved in the Norwegian live poultry genebank. Acta Agriculturae Scand Sect — Anim Sci. 2020;69:68–80.

[20] Heumann-Kiesler C, Sommerfeld V, Iffland H, Bennewitz J, Rodehutscord M, Hasselmann M. Insights into the mitochondrial and nuclear genome diversity of two high yielding strains of laying hens. Anim (Basel). 2021;11:825. 10.3390/ani11030825 PMC8001891 33804055

[21] Iqbal MA, Reyer H, Oster M, Hadlich F, Trakooljul N, Perdomo-Sabogal A, et al. Multi-omics reveals different strategies in the immune and metabolic systems of high-yielding strains of laying hens. Front Genet. 2022;13:858232. 35432452 10.3389/fgene.2022.858232 PMC9010826

[22] Roth C, Sims T, Rodehutscord M, Seifert J, Camarinha-Silva A. The active core microbiota of two high-yielding laying hen breeds fed with different levels of calcium and phosphorus. Front Physiol. 2022;13:951350. 36213242 10.3389/fphys.2022.951350 PMC9539745

[23] Reyer H, Oster M, Ponsuksili S, Trakooljul N, Omotoso AO, Iqbal MA, et al. Transcriptional responses in jejunum of two layer chicken strains following variations in dietary calcium and phosphorus levels. BMC Genom. 2021;22:485. 10.1186/s12864-021-07814-9 PMC8243909 34187361

[24] Omotoso AO, Reyer H, Oster M, Ponsuksili S, Trakooljul N, Muráni E, et al. Jejunal transcriptomic profiling of two layer strains throughout the entire production period. Sci Rep. 2021;11:20086. 34635722 10.1038/s41598-021-99566-5 PMC8505660

[25] Gonzalez-Uarquin F, Sommerfeld V, Rodehutscord M, Huber K. Interrelationship of myo-inositol pathways with systemic metabolic conditions in two strains of high-performance laying hens during their productive life span. Sci Rep. 2021;11:4641. 33633252 10.1038/s41598-021-84169-x PMC7907342

[26] Gesellschaft für Ernährungsphysiologie. Empfehlungen zur Energie-und Nährstoffversorgung der Legehennen und Masthühner. Frankfurt am Main: DLG-Verl. ;; 1999. (Energie- und Nährstoffbedarf landwirtschaftlicher Nutztiere, 7).

[27] Verband Deutscher Landwirtschaftlicher Untersuchungs- und Forschungsanstalten (VDLUFA). Handbuch der landwirtschaftlichen Versuchs und Untersuchungsmethodik (VDLUFA-methodenbuch), vol. III: Die Chemische Untersuchung von Futtermitteln. 1st ed. Darmstadt: VDLUFA; 2007.

[28] Boguhn J, Baumgärtel T, Dieckmann A, Rodehutscord M. Determination of titanium dioxide supplements in different matrices using two methods involving photometer and inductively coupled plasma optical emission spectrometer measurements. Arch Anim Nutr. 2009;63:337–42. 26967702 10.1080/17450390903052623

[29] Zeller E, Schollenberger M, Kühn I, Rodehutscord M. Hydrolysis of phytate and formation of inositol phosphate isomers without or with supplemented phytases in different segments of the digestive tract of broilers. J Nutr Sci. 2015;4:e1. 26090091 10.1017/jns.2014.62 PMC4463934

[30] Browning BL, Zhou Y, Browning S. R. A one-penny imputed genome from next-generation reference panels. Am J Hum Genet. 2018:103(3):338–48. 10.1016/j.ajhg.2018.07.015 PMC6128308 30100085

[31] Browning BL, Tian X, Zhou Y, Browning SR. Fast two-stage phasing of large-scale sequence data. Am J Hum Genet. 2021;108(10):1880–90. 10.1016/j.ajhg.2021.08.005 PMC8551421 34478634

[32] Chang CC, Chow CC, Tellier LC, Vattikuti S, Purcell SM, Lee JJ. Second-generation PLINK: rising to the challenge of larger and richer datasets. Gigascience. 2015;4:7. 25722852 10.1186/s13742-015-0047-8 PMC4342193

[33] Jolliffe I. Principal Component Analysis. In: Lovric M, editor. International encyclopedia of statistical science. Berlin: Springer; 2011. pp. 1094–6.

[34] Weir BS, Cockerham CC. Estimating F-statistics for the analysis of population structure. Evolution. 1984;38:1358–70. 28563791 10.1111/j.1558-5646.1984.tb05657.x

[35] Ripley B, Venables B, Bates DM, Hornik K, Gebhardt A, Firth D. Package ‘MASS’. 2013:113–20.

[36] Box GEP, Cox DR. An analysis of transformations. J R Stat Soc B: Stat Methodol. 1964;26:211–43.

[37] R Core Team. R: A language and environment for statistical computing. Vienna: R Foundation for Statistical Computing; 2021.

[38] Butler DG, Cullis BR, Gilmour AR, Gogel BJ, Thompson R. Fits the linear mixed model: ASReml-R Package Version 4.1.0.160. Hemel Hempstead: VSN International Ltd.; 2017.

[39] VanRaden PM. Efficient methods to compute genomic predictions. J Dairy Sci. 2008;91:4414–23. 18946147 10.3168/jds.2007-0980

[40] Butler DG, Cullis BR, Gilmour AR, Gogel BJ, Thompson R. ASReml-R reference manual version 4.2. VSN International Ltd.: Hemel Hempstead;; 2023.

[41] Iqbal MA, Hadlich F, Reyer H, Oster M, Trakooljul N, Murani E, et al. RNA-Seq-based discovery of genetic variants and allele-specific expression of two layer lines and broiler chicken. Evol Appl. 2023;16:1135–53. 37360029 10.1111/eva.13557 PMC10286233

[42] Schmucker S, Hofmann T, Sommerfeld V, Huber K, Rodehutscord M, Stefanski V. Immune parameters in two different laying hen strains during five production periods. Poult Sci. 2021;100:101408. 34530229 10.1016/j.psj.2021.101408 PMC8450256

[43] Oster M, Qasir H, Reyer H, Ponsuksili S, Trakooljul N, Sommerfeld V, et al. Transcriptional and endocrine orchestration of medullary bone formation and mineral turn-over in female chickens. Poult Sci. 2025;104:105798. 40961770 10.1016/j.psj.2025.105798 PMC12466242

[44] Silversides FG, Singh R, Cheng KM, Korver DR. Comparison of bones of 4 strains of laying hens kept in conventional cages and floor pens. Poult Sci. 2012;91:1–7. 22184423 10.3382/ps.2011-01453

[45] Kjaer J. Diurnal rhythm of feather pecking behaviour and condition of integument in four strains of loose housed laying hens. Appl Anim Behav Sci. 2000;65:331–47.

[46] Singh R, Cheng KM, Silversides FG. Production performance and egg quality of four strains of laying hens kept in conventional cages and floor pens. Poult Sci. 2009;88:256–64. 19151338 10.3382/ps.2008-00237

[47] Villanueva S, Ali ABA, Campbell DLM, Siegford JM. Nest use and patterns of egg laying and damage by 4 strains of laying hens in an aviary system. Poult Sci. 2017;96:3011–20. 28431049 10.3382/ps/pex104 PMC5850654

[48] Habig C, Distl O. Evaluation of bone strength, keel bone status, plumage condition and egg quality of two layer lines kept in small group housing systems. Br Poult Sci. 2013;54:413–24. 23906215 10.1080/00071668.2013.792405

[49] Habig C, Geffers R, Distl O. Differential gene expression from genome-wide microarray analyses distinguishes Lohmann Selected Leghorn and Lohmann Brown layers. PLoS ONE. 2012;7:e46787. 23056453 10.1371/journal.pone.0046787 PMC3466173

[50] Li X, Zhang D, Bryden WL. Calcium and phosphorus metabolism and nutrition of poultry: are current diets formulated in excess? Anim Prod Sci. 2017;57:2304.

[51] Sommerfeld V, Bennewitz J, Camarinha-Silva A, Feger M, Föller M, Huber K, et al. Effects of feeding diets without mineral P supplement on intestinal phytate degradation, blood concentrations of Ca and P, and excretion of Ca and P in two laying hen strains before and after onset of laying activity. Poult Sci. 2024;103:104407. 39489035 10.1016/j.psj.2024.104407 PMC11566335

