# Supplementary material for: Quantitative genetic analyses of traits related to calcium and phosphorus metabolism in two different laying hen strains
Source: Genet Sel Evol. 2026 Jul 21;58:35. doi: 10.1186/s12711-026-01066-5 (PMC13390245; doi:10.1186/s12711-026-01066-5)
Supplement: Supplementary file 1 — Supplementary Material 1: Additional file 1: Table S1. Ingredients and calculated composition of the experimental diet, Table S2. Analyzed composition of the experimental diet, Table S2. Analyzed composition of the experimental diet, Table S3. Box-Cox transformation lambda values of the analyzed traits in Lohmann Selected Leghorn (LSL) and Lohmann Brown (LB), Table S4. Fixed model effects for each trait in the Lohmann Selected Leghorn (LSL) strain, Table S5. Fixed model effects for each trait in the Lohmann Brown (LB) strain. [file 12711_2026_1066_MOESM1_ESM.docx]

**Table S1 Ingredients and calculated composition of the experimental diet**

| Ingredients, g/kg |  |
| --- | --- |
| Corn | 597.1 |
| Soybean meal | 256.0 |
| Alfalfa meal | 30.0 |
| Soybean oil | 15.0 |
| DL-Methionine | 4.5 |
| L-Lysine sulphate | 0.9 |
| Limestone, fine | 25.2 |
| Limestone, coarse | 58.0 |
| Sodium chloride | 2.8 |
| Choline chloride | 1.0 |
| Sodium bicarbonate | 2.0 |
| Vitamin mix^a^ | 2.0 |
| Mineral mix^b^ | 0.5 |
| TiO_2_ | 5.0 |
|  |  |
| Calculated, g/kg |  |
| Phosphorus (P) | 3.0 |
| Non-phytate-P | 1.2 |
| Calcium | 35.0 |
| Crude protein | 166.0 |
| Metabolizable energy, MJ/kg | 11.4 |

^a^Vitamin premix (Miavit GmbH, Essen, Germany), provided per kg of the complete diet: 10,000 IU vitamin A, 3,000 IU vitamin D3, 30 mg vitamin E, 2.4 mg vitamin K3, 100 mcg biotin, 1 mg folic acid, 3 mg vitamin B1, 6 mg vitamin B2, 6 mg vitamin B6, 30 mcg vitamin B12, 50 mg nicotinamide, 14 mg calcium-D-pantothenate.

^b^Trace element premix (Gelamin Gesellschaft für Tierernährung mbH, Memmingen, Germany), provided per kg of complete diet: 80 mg manganese from manganese-(II)-oxide, 60 mg zinc from zinc sulfate monohydrate, 25 mg iron from ferrous-(II)-sulfate monohydrate, 7.5 mg copper from cupric-(II)-sulfate pentahydrate, 0.6 mg iodine from calcium iodate, 0.2 mg selenium from sodium selenite.

**Table S2 Analyzed composition of the experimental diet**

| g/kg DM^a^ |  |
| --- | --- |
| P | 3.6 |
| Ca | 35.1 |
| Crude protein | 185 |
| *myo*-inositol | 0.2 |
| Ins(1,2,4,5,6)P_5_ | 0.4 |
| InsP_6_ | 6.9 |
| InsP_6_-P | 1.9 |
| Non phytate-P (calculated) | 1.7 |
| Ti^b^ | 3.0 |
| DM^a^, % | 90.8 |

^a^ *DM* dry matter; ^b^*Ti* titanium

**Table S3 Box-Cox transformation lambda values of the analyzed traits in Lohmann Selected Leghorn (LSL) and Lohmann Brown (LB)**

| Trait^a^ | LSL | LB |
| --- | --- | --- |
| NoE | 8.48 | 6.57 |
| AEW | 1.60 | 0.38 |
| ADFI | 1.87 | 2.39 |
| Ca_ex_ | -0.04 | 0.10 |
| CaR_ex_ | 3.06 | 2.42 |
| CaR_ex+eggs_ | 1.36 | 1.32 |
| P_ex_ | -0.76 | 0.79 |
| PR_ex_ | 1.16 | 1.33 |
| PR_ex+eggs_ | 1.12 | 1.31 |
| Ca_pl_ | 0.48 | 0.50 |
| Ca_il_ | -0.08 | -0.46 |
| P_pl_ | 0.76 | 0.05 |
| P_il_ | 0.30 | 1.07 |
| MI_egg_ | -0.32 | -0.18 |
| MI_pl_ | 0.84 | -0.31 |
| MI_il_ | -0.11 | -0.03 |
| InsP6_il_ | 1.22 | 1.14 |

^a^Full trait descriptions are given in Table 1

**Table S4 Fixed model effects for each trait in the Lohmann Selected Leghorn (LSL) strain**

| Trait^a^ | cohort | unit | EW_oviduct_ | NoE_int_ | NoE_int_^2^ | NoE_con_ | NoE_con_² | NoE_def_ | NoE_def_² | |
| --- | --- | --- | --- | --- | --- | --- | --- | --- | --- | --- |
| NoE | < 0.01* | 0.24 | n.m.^b^ | n.m.^b^ | n.m.^b^ | n.m.^b^ | n.m.^b^ | n.m.^b^ | n.m.^b^ | |
| AEW | < 0.01* | 0.38 | n.m.^b^ | < 0.01* | 0.01* | 0.16 | 0.95 | 0.74 | 0.36 | |
| ADFI | < 0.01* | 0.73 | n.m.^b^ | < 0.01* | 0.25 | 0.97 | 0.71 | 0.05* | 0.46 | |
| Ca_ex_ | < 0.01* | 0.01* | n.m.^b^ | 0.03* | 0.44 | 0.12 | 0.07 | 0.90 | 0.75 | |
| CaR_ex_ | < 0.01* | < 0.01* | n.m.^b^ | 0.05* | 0.17 | 0.16 | 0.07 | 0.62 | 0.64 | |
| CaR_ex+eggs_ | < 0.01* | < 0.01* | n.m.^b^ | 0.94 | 0.57 | 0.82 | 0.61 | 0.08 | 0.91 | |
| P_ex_ | < 0.01* | 0.14 | n.m.^b^ | 0.12 | 0.30 | 0.27 | 0.60 | 0.23 | 0.63 | |
| PR_ex_ | < 0.01* | 0.07 | n.m.^b^ | 0.83 | 0.20 | 0.93 | 0.47 | 0.15 | 0.82 | |
| PR_ex+eggs_ | < 0.01* | 0.04* | n.m.^b^ | 0.16 | 0.55 | 0.26 | 0.91 | 0.02* | 0.68 | |
| Ca_pl_ | 0.10 | n.m.^b^ | 0.66 | 0.02* | 0.04* | 0.05* | 0.48 | 0.61 | 0.44 | |
| Ca_il_ | 0.01* | n.m.^b^ | 0.68 | 0.85 | 0.75 | 0.19 | 0.23 | 0.51 | 0.59 | |
| P_pl_ | 0.01* | n.m.^b^ | < 0.01* | 0.46 | 0.06 | 0.21 | 0.58 | 0.62 | 0.08 | |
| P_il_ | < 0.01* | n.m.^b^ | < 0.01* | 0.70 | 0.65 | 0.11 | 0.44 | 0.33 | 0.47 | |
| MI_egg_ | < 0.01* | 0.31 | n.m.^b^ | 0.02* | 0.06 | 0.72 | 0.18 | 0.29 | 0.83 | |
| MI_pl_ | 0.53 | n.m.^b^ | < 0.01* | 0.02* | 0.76 | 0.77 | >0.99 | 0.69 | 0.92 | |
| MI_il_ | < 0.01* | n.m.^b^ | 0.03* | 0.03* | 0.76 | 0.84 | 0.51 | 0.80 | 0.86 | |
| InsP6_il_ | < 0.01* | n.m.^b^ | < 0.01* | 0.39 | 0.34 | 0.05* | 0.24 | 0.53 | 0.41 | |
| Conditional Wald tests were performed to test the cohort, unit, egg stage in oviduct at slaughter (EW_oviduct_), number of intact (NoE_int_), number of consumed (NoE_con_) and number of defective eggs (NoE_def_) effects of the analyzed traits. Unit (EW_oviduct_) was not modeled (n.m.) for the traits analyzed at slaughter (during excreta sampling period in live animals). The number of intact, consumed and defective eggs was modeled as linear and quadratic regression variable. ^a^Full trait descriptions are given in Table 1; ^b^not modeled; *significant (p ≤ 0.05) fixed effects | | | | | | | | | |  |

**Table S5 Fixed model effects for each trait in the Lohmann Brown (LB) strain**

| Trait^a^ | cohort | unit | EW_oviduct_ | NoE_int_ | NoE_int_^2^ | NoE_con_ | NoE_con_² | NoE_def_ | NoE_def_² |
| --- | --- | --- | --- | --- | --- | --- | --- | --- | --- |
| NoE | < 0.01* | 0.83 | n.m.^b^ | n.m.^b^ | n.m.^b^ | n.m.^b^ | n.m.^b^ | n.m.^b^ | n.m.^b^ |
| AEW | < 0.01* | 0.59 | n.m.^b^ | 0.06 | 0.02* | 0.06 | 0.96 | 0.03* | 0.56 |
| ADFI | < 0.01* | 0.01* | n.m.^b^ | 0.01* | 0.82 | 0.47 | 0.01* | 0.09 | 0.27 |
| Ca_ex_ | 0.08 | > 0.99 | n.m.^b^ | < 0.01* | 0.14 | 0.51 | 0.09 | < 0.01* | 0.84 |
| CaR_ex_ | < 0.01* | 0.27 | n.m.^b^ | < 0.01* | 0.10 | 0.50 | 0.01* | 0.01* | 0.92 |
| CaR_ex+eggs_ | < 0.01* | < 0.01* | n.m.^b^ | 0.23 | 0.01* | 0.37 | 0.26 | 0.32 | 0.86 |
| P_ex_ | < 0.01* | 0.53 | n.m.^b^ | 0.01* | 0.01* | 0.66 | 0.04* | 0.01* | 0.26 |
| PR_ex_ | < 0.01* | 0.01* | n.m.^b^ | 0.92 | 0.28 | 0.58 | 0.16 | 0.04* | 0.15 |
| PR_ex+eggs_ | < 0.01* | < 0.01* | n.m.^b^ | 0.04* | 0.84 | 0.56 | 0.25 | < 0.01* | 0.17 |
| Ca_pl_ | 0.92 | n.m.^b^ | 0.02* | 0.37 | 0.73 | 0.93 | 0.38 | 0.83 | 0.37 |
| Ca_il_ | 0.06 | n.m.^b^ | 0.74 | 0.26 | 0.91 | 0.35 | 0.70 | 0.21 | 0.18 |
| P_pl_ | 0.97 | n.m.^b^ | 0.31 | 0.59 | 0.30 | 0.66 | 0.09 | 0.07 | 0.05* |
| P_il_ | < 0.01* | n.m.^b^ | < 0.01* | 0.48 | 0.32 | 0.79 | 0.08 | 0.42 | 0.71 |
| MI_egg_ | < 0.01* | 0.04* | n.m.^b^ | 0.11 | 0.53 | 0.07 | 0.56 | 0.85 | 0.44 |
| MI_pl_ | 0.36 | n.m.^b^ | 0.83 | 0.03* | 0.75 | 0.21 | 0.34 | 0.84 | 0.95 |
| MI_il_ | < 0.01* | n.m.^b^ | 0.25 | 0.23 | 0.42 | 0.49 | 0.50 | 0.98 | 0.14 |
| InsP6_il_ | < 0.01* | n.m.^b^ | < 0.01* | 0.19 | 0.64 | 0.60 | 0.14 | 0.18 | 0.45 |
| Conditional Wald tests were performed to test the cohort, unit, egg stage in oviduct at slaughter (EW_oviduct_), number of intact (NoE_int_), number of consumed (NoE_con_) and number of defective eggs (NoE_def_) effects of the analyzed traits. Unit (EW_oviduct_) was not modeled (n.m.) for the traits analyzed at slaughter (during excreta sampling period in live animals). The number of intact, consumed and defective eggs was modeled as linear and quadratic regression variable. ^a^Full trait descriptions are given in Table 1; ^b^not modeled; *significant (p ≤ 0.05) fixed effects | | | | | | | | | |
